# Duration of hemodynamic effects of crystalloids in patients with circulatory shock after initial resuscitation

**DOI:** 10.1186/s13613-014-0025-9

**Published:** 2014-08-01

**Authors:** Thieme Souza Oliveira Nunes, Renata Teixeira Ladeira, Antônio Tonete Bafi, Luciano Cesar Pontes de Azevedo, Flavia Ribeiro Machado, Flávio Geraldo Rezende Freitas

**Affiliations:** 1 Disciplina de Anestesiologia, Dor e Terapia Intensiva, Universidade Federal de São Paulo, Rua Napoleão de Barros 715 - 5° andar, São Paulo SP 04024-900, Brazil

**Keywords:** Fluid, Fluid responsiveness, Fluid resuscitation, Crystalloids, Circulatory shock, Hemodynamics

## Abstract

**Background:**

In the later stages of circulatory shock, monitoring should help to avoid fluid overload. In this setting, volume expansion is ideally indicated only for patients in whom the cardiac index (CI) is expected to increase. Crystalloids are usually the choice for fluid replacement. As previous studies evaluating the hemodynamic effect of crystalloids have not distinguished responders from non-responders, the present study was designed to evaluate the duration of the hemodynamic effects of crystalloids according to the fluid responsiveness status.

**Methods:**

This is a prospective observational study conducted after the initial resuscitation phase of circulatory shock (>6 h vasopressor use). Critically ill, sedated adult patients monitored with a pulmonary artery catheter who received a fluid challenge with crystalloids (500 mL infused over 30 min) were included. Hemodynamic variables were measured at baseline (T0) and at 30 min (T1), 60 min (T2), and 90 min (T3) after a fluid bolus, totaling 90 min of observation. The patients were analyzed according to their fluid responsiveness status (responders with CI increase >15% and non-responders ≤15% at T1). The data were analyzed by repeated measures of analysis of variance.

**Results:**

Twenty patients were included, 14 of whom had septic shock. Overall, volume expansion significantly increased the CI: 3.03 ± 0.64 L/min/m^2^ to 3.58 ± 0.66 L/min/m^2^ (*p* < 0.05). From this period, there was a progressive decrease: 3.23 ± 0.65 L/min/m^2^ (*p* < 0.05, T2 versus T1) and 3.12 ± 0.64 L/min/m^2^ (*p* < 0.05, period T3 versus T1). Similar behavior was observed in responders (13 patients), 2.84 ± 0.61 L/min/m^2^ to 3.57 ± 0.65 L/min/m^2^ (*p* < 0.05) with volume expansion, followed by a decrease, 3.19 ± 0.69 L/min/m^2^ (*p* < 0.05, T2 versus T1) and 3.06 ± 0.70 L/min/m^2^ (*p* < 0.05, T3 versus T1). Blood pressure and cardiac filling pressures also decreased significantly after T1 with similar findings in both responders and non-responders.

**Conclusions:**

The results suggest that volume expansion with crystalloids in patients with circulatory shock after the initial resuscitation has limited success, even in responders.

## Background

Intravascular fluid administration is the first-line intervention for the restoration of hemodynamic stability in critically ill patients [1]. The expected response to fluid infusion is an increase in venous return leading to an augmentation of cardiac output (CO) through the Frank-Starling mechanism [2]. However, only half of all hemodynamically unstable patients are preload-responsive [3]. This phenomenon could be particularly important in patients who are in the intensive care unit (ICU) for several hours or days after the initial resuscitation. At this stage, especially if a lung is injured, positive fluid balance becomes a major concern. In this case, it is recommended to use predictors of fluid responsiveness to distinguish between patients who can benefit from volume expansion (responders) and those in whom fluid loading can be futile and even deleterious (non-responders) [4].

However, even in responders, using fluids after the initial resuscitation to treat hypotension or indices of inadequate tissue perfusion could be inappropriate. Two factors justify this concern: First, fluid administration can lead to an increased fluid balance, which is associated with a worse outcome in ICU patients [5],[6]. Second, isotonic crystalloid solutions, usually the choice for fluid replacement, have a limited effect on plasma volume. In theory, after a short period of balance, only approximately 20% of an intravenously infused crystalloid solution will remain in the intravascular space to support plasma volume [7].

Studies designed to examine fluid responsiveness only address the immediate hemodynamic response to a fluid challenge. Moreover, previous studies evaluating the hemodynamic effect of crystalloids have not distinguished responders from non-responders. The present study aimed to evaluate the short-term hemodynamic effects of volume expansion with crystalloids after the initial resuscitation of circulatory shock in patients receiving vasopressors and to compare these effects in responders and non-responders.

## Methods

This observational study was conducted in a 35-bed mixed ICU in a university hospital. The institutional Research and Ethics Committee (Universidade Federal de São Paulo) approved the study, and informed consent was waived due to its purely observational nature.

We prospectively included adult patients with circulatory shock who received one fluid challenge with crystalloids (Ringer's lactate or sodium chloride 0.9% solution, 500 mL infused over 30 min) indicated for inadequate tissue perfusion by the attending physician. Only patients treated with vasopressors for at least 6 h, under mechanical ventilation, receiving continuous sedation (Ramsay score 5 to 6), and monitored with an arterial catheter and a pulmonary artery catheter (Edwards Lifesciences, Irvine, CA, USA) were eligible for study inclusion. We excluded patients in whom the primary cause of hypotension was active bleeding (suspected or confirmed), burn injury, and cardiogenic shock, defined as cardiac index (CI) <1.8 L/min/m^2^ without support and pulmonary artery occlusion pressure ≥18 mmHg.

The patients were followed during fluid infusion (30 min) and for 60 min after. Throughout this observational period, if the attending physician changed ventilator parameters, doses of the sedative, inotropic and vasopressor medications or administered a new volume expansion, the patient was excluded from the analysis.

As part of the routine care, we registered a complete set of hemodynamic and respiratory measurements, including arterial and mixed venous blood gases, and hemoglobin and arterial lactate levels at the beginning (T0) and end of the volume expansion (T1). Only hemodynamic measurements were registered at 60 min (T2) and 90 min (T3). Thus, the study has four different time points: baseline (T0), 30 min (T1), 60 min (T2), and 90 min (T3) after the fluid bolus, totaling 90 min of observation. Patients who did not have laboratory samples collected before and after the fluid infusion were excluded from the analysis.

The CI was measured using a semi-continuous thermodilution technique considering the average value of four consecutive measurements from the STAT mode screen of the Vigilance® monitor (Edwards Lifesciences, Irvine, CA, USA). We also measured the pulse pressure variation (PPV) in patients without limitations to this assessment, with a multiparameter bedside monitor (DX 2020, Dixtal, São Paulo, Brazil) using the automatic method [8],[9]. All pressures were determined using the cursor line of the bedside monitor screen. The reference point for right atrial pressure (RAP) and pulmonary artery occlusion pressure (PAOP) was the base of the ‘a’ wave at the end-expiration phase with the zero reference level settled at the mid-chest and the head of the bed elevated at approximately 30°.

### Statistical analysis

Data are expressed as numbers (%), means ± standard deviation (SD), or medians and interquartile ranges (25th to 75th percentile), as appropriate. Changes in CI after the fluid infusion were expressed as percentages. The distribution of continuous variables was assessed by the Shapiro-Wilk test.

A one-way repeated measures analysis of variance (ANOVA) was used to compare the hemodynamic variables in different time points (T0, T1, T2, and T3). Second, the patients were divided into two subgroups according to the percent increase in CI in response to volume expansion: ‘responders’ had a CI increase of at least 15%, whereas ‘non-responders’ had a CI increase of less than 15%. A two-way repeated measure ANOVA (to include a subgroup interaction effect) was also conducted. We used Bonferroni adjustment for multiple comparisons.

When only measurements at T0 and T1 were available (hemoglobin, lactate, and mixed venous oxygen saturation), we used Mann-Whitney *U* test or a *t* test to compare responders and non-responders at baseline and Wilcoxon’s rank-sum test or a paired *t* test to assess the effects of intravascular volume expansion.

We used SPSS version 17.0 for Windows (SPSS Inc., Chicago, IL, USA). The results with *p* values of <0.05 were considered to be significant.

## Results

**Figure 1:**
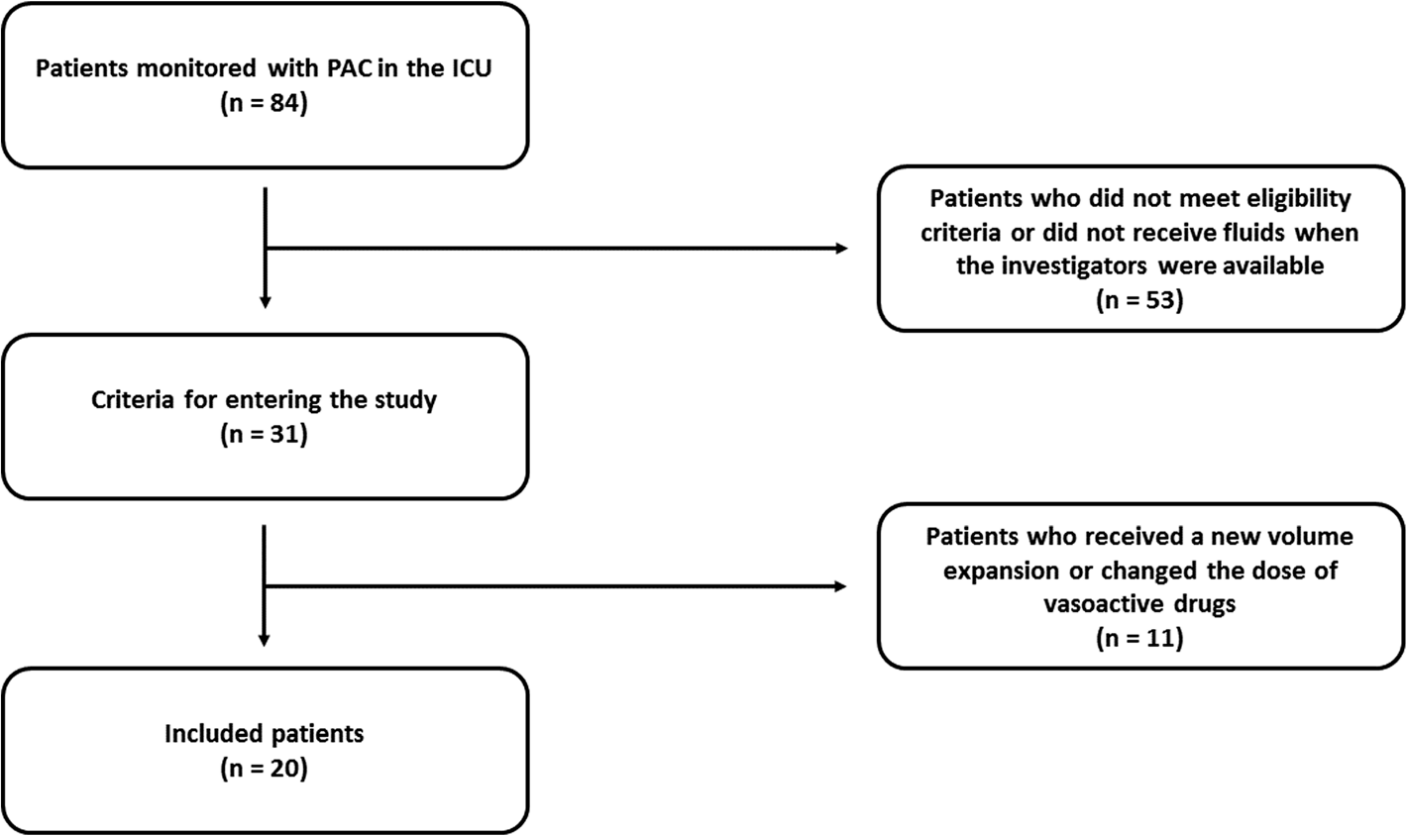
**Study flowchart.** PAC, pulmonary artery catheter; ICU, intensive care unit.

From September 2011 to January 2013, 84 patients with circulatory shock were monitored with a pulmonary artery catheter in the ICU, of whom 20 patients were included (Figure 1).

The circulatory shock was secondary to sepsis (*n* = 14), major surgery (*n* = 5), or multiple trauma (*n* = 1). The median duration of vasopressor use before inclusion was 25 (19.5 to 34.0) h. The fluid balance from the time when vasopressor treatment was initiated to volume expansion was 7,217 (6,345 to 8,545) mL. There was no difference between responders and non-responders regarding the duration of shock (*p* = 0.59) and fluid balance (*p* = 0.81). Patient characteristics are listed in Table 1.

**Table 1:** Patient characteristics at baseline

**Patient characteristic**	**Data**
Age (years)	63.7 ± 14.2
Gender [male (%)]	16 (80)
APACHE II	21.50 (18.0 to 26.0)
SOFA	10.5 (9.0 to 12.0)
PEEP (cm H_2_O)	8.0 (5.0 to 10.0)
FiO_2_ (%)	40 (40 to 50)
Tidal volume (mL)	487 ± 95.1
Cstat (mL/cm H_2_O)	39.2 ± 13.5
PaO_2_/FiO_2_	206.6 ± 72.9
Time of vasopressor use (h)	25.0 (19.5 to 34.0)
Fluid balance (mL)^a^	7,217 (6,345 to 8,545)
Causes of shock	
Sepsis [*n* (%)]	14 (70)
Major surgery [*n* (%)]	5 (25)
Multiple trauma [*n* (%)]	1 (5)
Catecholamine infusion	
Norepinephrine, μg/kg/min (*n* = 20)	0.33 (0.21 to 0.83)
Epinephrine, μg/kg/min (*n* = 5)	0.26 (0.14 to 0.35)
Dobutamine, μg/kg/min (*n* = 5)	6.0 (5.1 to 7.1)
Survivors (ICU) [*n* (%)]	7 (35)

APACHE II, Acute Physiology and Chronic Health Evaluation II; SOFA, Sequential Organ Failure Assessment score; PEEP, positive end-expiratory pressure; FiO_2_, fraction of inspired oxygen; Cstat, static respiratory compliance; PaO_2_, partial pressure of oxygen; ICU, intensive care unit. ^a^From shock to fluid infusion. Quantitative variables are expressed as means (SD) or medians (IQR 25th to 75th percentile).

The indications for volume expansion were hyperlactatemia (30%), hypotension (20%), clinical signs of poor skin perfusion (20%), reduce vasopressors (20%), and oliguria (10%). The diuresis during study period was 37.50 (0.00 to 97.50) mL, with no differences between responders and non-responders (*p* = 0.77).

**Figure 2:**
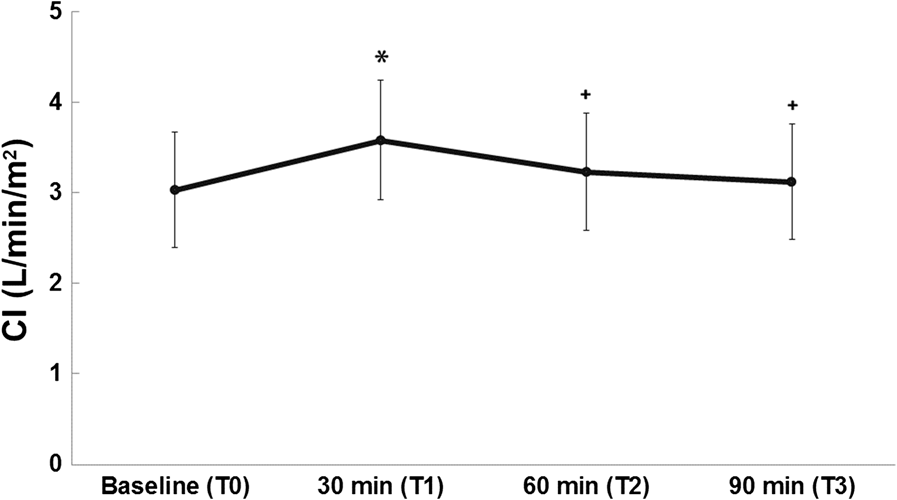
**Cardiac index.** CI, cardiac index. Baseline, 3.03 ± 0.64; T1, 3.58 ± 0.66; T2, 3.23 ± 0.65; T3, 3.12 ± 0.64. *p < 0.05 versus baseline, ^+^*p* < 0.05 versus T1.

Overall, CI increased significantly after volume expansion; however, in T3, CI decreased to the baseline values (Figure 2). Cardiac filling pressures and mean arterial pressure (MAP) also increased significantly in T1 and decreased after volume expansion (T2 and T3) (Table 2).

**Table 2:** Time-course of hemodynamic variables (all patients)

**Variables**	**T0 (baseline)**	**T1 (30 min)**	**T2 (60 min)**	**T3 (90 min)**
RAP (mmHg)	8.20 ± 3.02	10.5 ± 3.17*	9.0 ± 3.06*^,+^	9.10 ± 4.00*^,+^
PAOP (mmHg)	8.52 ± 3.16	10.13 ± 3.71*	8.81 ± 3.15^+^	8.67 ± 3.36^+^
MAP (mmHg)	73.70 ± 8.18	81.70 ± 9.64*	77.95 ± 10.28*	77.60 ± 13.05
HR (beats/min)	109 ± 18.48	106.15 ± 18.11	107.35 ± 19.20	107.55 ± 19.45
SvO_2_ (%)	66.30 ± 9.03	69.07 ± 7.98*		
Lactate (mg/dL)	3.44 (1.92 to 5.47)	3.0 (1.86 to 5.53)		
Hb (g/dL)	9.59 ± 1.62	9.17 ± 1.80		

RAP, right atrial pressure; PAOP, pulmonary artery occlusion pressure; MAP, mean arterial pressure; HR, heart rate; SvO_2_, mixed venous oxygen saturation; Hb, hemoglobin. *p < 0.05 versus baseline, ^+^*p* < 0.05 versus T1. Data are expressed as means (SD) or medians (IQR 25th to 75th percentile).

Thirteen patients were considered to be volume responders, as their CI increased more than 15%. CI did not change over time in non-responders group, whereas in responders CI changed significantly: after an initial increase from T0 to T1, there was a reduction in T2 and T3. Cardiac filling pressures and MAP were similar between responders and non-responders over time. These variables decreased after volume expansion (T2 and T3) toward baseline values. Responders and non-responders showed no significant change in heart rate (HR) over time (Table 3).

**Table 3:** Time-course of hemodynamic variables according to fluid responsiveness status

**Variable**		**T0 (baseline)**	**T1 (30 min)**	**T2 (60 min)**	**T3 (90 min)**
CI (L/min/m^2^)	R	2.84 ± 0.61	3.57 ± 0.65*	3.19 ± 0.69*^,+^	3.06 ± 0.70^+^
	NR	3.40 ± 0.55	3.59 ± 0.74	3.31 ± 0.61	3.24 ± 0.55
RAP (mmHg)	R	8.54 ± 3.36	10.23 ± 3.72*	9.08 ± 3.59*^,+^	9.23 ± 3.54*^,+^
	NR	7.57 ± 2.37	9.71 ± 1.98*	8.86 ± 1.95*^,+^	8.86 ± 2.27*^,+^
PAOP (mmHg)	R	8.77 ± 3.30	10.64 ± 4.03*	8.69 ± 3.2	8.89 ± 3.62
	NR	8.04 ± 3.08	9.17 ± 3.06*	9.01 ± 3.30	8.26 ± 3.05
MAP (mmHg)	R	74.62 ± 6.84	83.92 ± 8.20*	79.62 ± 10.94	79.77 ± 14.02
	NR	72.00 ± 10.63	77.57 ± 11.36*	74.86 ± 8.84	73.57 ± 10.83
HR (beats/min)	R	107.54 ± 19.83	105.77 ± 19.63	107.85 ± 20.43	108.62 ± 21.06
	NR	111.71 ± 16.80	106.86 ± 16.31	106.43 ± 18.17	105.57 ± 17.42
Lactate (mmol/L)	R	2.78 (1.50 to 6.89)	2.78 (1.56 to 6.72)		
	NR	3.44 (2.22 to 4.22)	3.44 (2.22 to 5.11)		
Hb (g/dL)	R	9.36 ± 1.76	9.34 ± 2.06		
	NR	10.01 ± 1.36	8.83 ± 1.26*		
SvO_2_ (%)	R	63.00 ± 9.08	66.30 ± 8.30*		
	NR	72.46 ± 5.02	74.20 ± 4.01		

R, responders; NR, non-responders; CI, cardiac index; RAP, right atrial pressure; PAOP, pulmonary artery occlusion pressure; MAP, mean arterial pressure; HR, heart rate; SvO_2_, mixed venous oxygen saturation; Hb, hemoglobin. The groups (R and NR) showed significant CI difference over time (*p* = 0.009). The CI did not change over time in NR (*p* = 0,306). The groups (R and NR) showed no significant difference over time in RAP, PAOP, MAP, and HR (*p* > 0.05). Mean values of RAP, PAOP, MAP, and HR were similar between R and NR at T0, T1, T2, and T3 (*p* > 0.05). The groups (R and NR) showed significant difference in SvO_2_ (*p* = 0.02) at T0. The groups (R and NR) showed no significant difference in lactate levels (*p* = 0.72) and Hb (*p* = 0.46) at T0. *p < 0.05 versus baseline, ^+^*p* < 0.05 versus T1. Data are expressed as means (SD) or medians (IQR 25th to 75th percentile).

All patients were in sinus rhythm, and only one had ventricular extrasystoles not allowing automatic PPV measurement. We also did not record PPV in other 14 patients because of spontaneous ventilation or ventilation with low tidal volume. Thus, PPV could be registered only in five patients. The four responders at baseline were not fluid-responsive at T1 (PPV < 12%).

## Discussion

The main finding of our study was that CI decreased toward baseline values 60 min after fluid infusion. Even in responders, the hemodynamic changes nearly disappeared in this period. The results strengthen the body of evidence that shows a short hemodynamic effect after volume expansion with crystalloids.

As there were no acute fluid losses, the main finding of our study has some possible explanations. First, crystalloids have limited intravascular volume effect, as suggested by volume kinetics studies of Ringer’s solution [10]–[13]. This has been demonstrated even in normal vascular endothelial conditions. A study in healthy volunteers demonstrated that 68% of the saline infused (1,000 mL over 60 min) had escaped from the intravascular to the extravascular space at the end of infusion, as estimated from hematocrit/hemoglobin changes [12]. According to another study, in healthy volunteers who received lactated Ringer’s solution (1,000 mL over 5 to 7 min) after blood withdrawal, the peak increase in the intravascular volume occurred immediately after completion of infusion (630 ± 127 mL), and the intravascular volume expanding effect declined rapidly after its initial peak (only 403 ± 88 mL by 15 min) [13]. Second, under inflammatory conditions, such as sepsis, surgery, or trauma, the damage of the endothelial glycocalyx decreases vascular barrier function and leads to protein extravasation [14],[15]. In these settings, the hemodynamic effects of crystalloid may be smaller. In postoperative hypovolemic patients, Ringer’s solution (10 mL/kg over 30 min) significantly improved hemodynamics at the end of volume loading, but this effect completely disappeared at 120 min [16]. One important point is that there might be different behaviors among our patients, depending on the origin of shock, the degree of vasodilation, the supposed degree of capillary leak, and the shock severity.

A number of experimental and clinical studies, generally comparing crystalloids with colloid solutions, have reached similar conclusions through different monitoring tools and endpoints [12],[13],[16]–[20]. In these studies, the hemodynamic effects of colloids lasted longer than those of crystalloids. In general, colloid administration restores hemodynamic stability more rapidly and with less volume in a variety of clinical conditions [17]–[20]. Despite theoretical benefits favoring colloids over crystalloids to achieve resuscitation endpoints, this advantage is not as clear in clinical application [21],[22]. Comparisons in patients with capillary leak show that the ratio between required volumes in the crystalloid and colloid is in a range between 1 and 2 (not threefold or higher ratio as recommended by textbooks) [21]. Moreover, there are concerns about the safety of synthetic colloids [23]–[26]. Thus, we chose to evaluate only the use of fluid challenges with crystalloids in this study.

Rather than increasing the plasma volume or reaching static parameters of preload, the main reason for intravascular fluid administration is to increase stroke volume. If the fluid challenge does not increase stroke volume, the volume loading is useless to the patient [1]. In our sample, volume expansion led to an immediate increase in cardiac filling pressures in all patients and significantly increased CI in a subset of them (responders). This result aligns with studies designed to examine fluid responsiveness [27]–[29]. However, these studies only describe the effect of volume expansion immediately at the end of infusion. We demonstrated a rapid CI reduction after this time point in all responders. RAP decreased after volume expansion as compared to T1 values. PAOP and MAP were similar to baseline values at T3. Our findings suggest that post-resuscitation volume expansion in responders with circulatory shock has limited impact.

Our study was performed after the resuscitation phase. It is unclear whether hemodynamic effects would be sustained for longer in the initial resuscitation phase. Hypotensive septic patients who received 5 mL/kg of normal saline over 15 min had a sustained increase in CO within 120 min [30]. However, experimental models of septic shock suggest different results [31],[32]. Our findings allow us to speculate that repeated volume expansions would be required in responders to sustain CO. Interestingly, studies performed in the context of goal-directed therapy in the perioperative period demonstrated that minimizing stroke volume variation by volume loading, and thus sustaining CO, was associated with increased fluid administration [33]–[36].

We can suggest that we should be more judicious in pursuing volume expansion in the post-resuscitation phase, even in responders. Excessive intravenous fluid therapy leading to positive fluid balances and interstitial edema could be associated with adverse outcomes in critically ill patients [37]–[40]. In contrast to hypodynamic circulation, as seen in early severe sepsis [41], late-phase patients usually have elevated RAP, CO, and mixed venous oxygen saturation (SvO_2_) [42],[43]. It has been suggested that the preferred use of vasoactive drugs may be an acceptable approach for such late-phase patients [39].

The current study has an important strength. In contrast with previous studies, we described the hemodynamic effects of volume expansion according to a fluid responsiveness status (CI response to volume expansion). Previous studies assessed these effects using labeling and dilutional methods or static preload parameters as endpoint, which might have limited applicability. Our study also had limitations. The first and main limitation was the sample size. A larger sample might have further strengthened our results. Second, the amount of infused fluid (500 mL of crystalloids over 30 min) might have not been enough to impact the CI [44]. However, in our study, there was a significant increase in RAP, and the fluid administration was able to significantly increase the CI in 13 patients. Moreover, in responders whom PPV was measured, the value fell below 12% in all. Third, we did not have hemoglobin/hematocrit values at different time points (T0, T1, T2, and T3) to calculate changes in blood volume, which could enhance our findings. Fourth, we could not fully assess cardiac function using echocardiography before and after fluid challenge to rule out the presence of previous cardiac dysfunction that could be aggravated by the circulatory shock. Finally, we use a semi-continuous thermodilution technique for determining the CO. This method may underestimate the changes in CO if it is measured immediately after the fluid infusion. As the patient was followed for 90 min, this limitation most likely did not affect the conclusions of the study.

## Conclusions

In patients with circulatory shock after an initial resuscitation, our findings suggest that the hemodynamic effects of crystalloid administration are not sustained after 60 min, even in responders.

## Abbreviations

ANOVA: analysis of variance

APACHE II: Acute Physiology and Chronic Health Evaluation II

CI: cardiac index

CO: cardiac output

Cstat: static respiratory compliance

FiO_2_: fraction of inspired oxygen

Hb: hemoglobin

HR: heart rate

ICU: intensive care unit

IQR: interquartile range

MAP: mean arterial pressure

PaO_2_: partial pressure of oxygen

PAOP: pulmonary artery occlusion pressure

PEEP: positive end-expiratory pressure

PPV: pulse pressure variation

RAP: right atrial pressure

SD: standard deviation

SOFA: Sequential Organ Failure Assessment score

SPSS: Statistical Package for the Social Sciences

SvO_2_: mixed venous oxygen saturation

## Competing interests

The authors declare that they have no competing interests.

## Authors’ contributions

FGRF, TSON, and RTL designed and coordinated the study. FGRF, TSON, and ATB contributed to data collection. FGRF and FRM drafted the manuscript. TSON, ATB, RTL, and LCPA revised the article. All authors read and approved the final manuscript.
